# 

**Published:** 1907-06-22

**Authors:** 


### General Practitioners' Contributions.

### Suggestions Invited.

The Editor will welcome suggestions for the establishment of any new section in The Hospital, and will be glad to supply information on any subject of interest or importance to members of the profession in any part of the world.

### Notices and Answers to Correspondence.

All MSS., letters, books for review, and other matters intended for the Editor, should be addressed to The Editor, The Hospital Building, 28 and 29 Southampton Street, Strand, London, W.C.

### Business Notices.

Letters relating to the Publishing, Sale and Advertisement Departments must be addressed to the Manager (*not to the Editor*) :—The Manager, The Hospital Building, 28 and 29 Southampton Street, Strand, London, W.C.

### Annual Subscriptions.

The rate of annual subscriptions to The Hospital, either direct from the Office or through agents, is :—

For the United Kingdom ... ... ... 13s. a year
To the Colonies and Abroad ... ... 17s. a year inclusive of postage in each case.

### Subscriptions.

Subscriptions are payable in advance. Cheques and Postal Orders should be made payable to :—

The Scientific Press, Ltd., and should be crossed: London and County Banking Co., Ltd.

